# A qualitative study to understand the experience of somatostatin analog treatments from the perspective of patients with neuroendocrine tumors

**DOI:** 10.1007/s00520-022-07054-x

**Published:** 2022-04-27

**Authors:** Caroline Seo, Erica Horodniceanu, Rachel Shah, Grace Goldstein, David Ray, Bonita Bennett, Alexandria Phan, Kelly McCarrier

**Affiliations:** 1 Pharmerit—an OPEN Health Company, 4350 East West Highway, Suite 1100, Bethesda, MD USA; 2 The Carcinoid Cancer Foundation, Mt. Kisco, NY USA; 3 Ipsen Biopharmaceuticals, Cambridge, MA USA; 4 Abramson Cancer Center, Philadelphia, PA USA; 5 UT Health East Texas North Campus MD Anderson Cancer Center, Tyler, TX USA

**Keywords:** Qualitative research, Neuroendocrine tumors, Somatostatin analogs, Patient experience, Patient preference

## Abstract

**Purpose:**

Neuroendocrine tumors (NETs) negatively impact patients’ quality of life. Octreotide long-acting release (LAR) and lanreotide depot are somatostatin analogs (SSAs) approved to treat NETs. The study objective was to explore SSA treatment experiences and preferences of patients with NETs.

**Methods:**

Qualitative interviews were conducted in US adults (≥ 21 years) with NETs who had ≥ 6 months’ treatment with each SSA and transitioned from octreotide LAR to lanreotide depot within the previous year. Participants were asked open-ended questions about their experiences with octreotide LAR and lanreotide depot, treatment preferences, and SSA treatment attributes.

**Results:**

Twenty participants (mean age: 58 years; 90% female; 85% white) completed interviews. The most common reasons for treatment transition were doctor recommendation (70%), treatment not working as expected (55%), and injection type preference (45%). Participants reported 34 unique favorable attributes of SSA treatment and 82 unique unfavorable attributes. Symptom control was the most frequently reported favorable attribute (associated with octreotide LAR by 60% of participants and lanreotide depot by 65%). Painful injection (65%) was most frequently cited unfavorable attribute for octreotide LAR and injection experience dependent on administrator (35%) for lanreotide depot. The three SSA treatment attributes rated as most important were side effects, symptom control, and ability to stabilize tumor*.*

**Conclusion:**

Our qualitative data provide valuable insight into the treatment attributes that patients with NETs consider important when making SSA treatment decisions. Factors related to injection administration, side effects, and symptom control are important to patients and should be included in patient-provider communications in clinical contexts.

**Supplementary Information:**

The online version contains supplementary material available at 10.1007/s00520-022-07054-x.

## Introduction

Neuroendocrine tumors (NETs) are a rare type of tumor that arise from cells of the diffuse endocrine system, rather than cells from a specific organ or tissue, making them biologically and clinically diverse, and are often detected when incurable in advanced stage. A subset of NETs, functional tumors, are able to oversecrete peptides and other biological substances normally secreted by neuroendocrine cells (e.g., serotonin, insulin, gastrin, and glucagon), resulting in characteristic hormonal syndromes (e.g., carcinoid syndrome, insulinoma) [1]. Although NETs can occur throughout the body, over half of NETs arise within the gastrointestinal tract and pancreas (i.e., gastroenteropancreatic [GEP] NETs) [2] and approximately 25% in the lung [3]. The primary tumor location, along with other factors (e.g., age, sex, histologic features, and stage at diagnosis), is also linked to prognosis in patients with NETs [4]. For example, in a recent analysis of US Surveillance, Epidemiology, and End Results (SEER) data, median overall survival for all patients with NETs was 9.3 years, but significant differences in median overall survival were observed between patients with lung NETs (5.5 years) and those with GEP NETs in locations such as the rectum (24.6 years) and appendix (> 30.0 years) [4].

The incidence and prevalence of NETs in the USA are rising and may be attributed to increased disease awareness and improved diagnostic technology, thereby leading to earlier disease detection and stage migration [4]. According to a study using the US SEER database, there has been a 6.4-fold increase in the age-adjusted incidence of NETs from the years 1973 to 2012 [4]. The 20-year limited-duration prevalence of NETs in the USA was estimated to be over 171,000 (as of January 2014) [4]. In addition, it is estimated that 19% of patients with NETs in the USA have carcinoid syndrome, representing an 8% increase from the years 2000 to 2011 [5].

Neuroendocrine tumors are associated with significant burden and have a detrimental influence on quality of life among affected individuals [6, 7]. Symptoms commonly reported among patients with NETs and/or carcinoid syndrome include pain, fatigue, diarrhea, flushing, abdominal discomfort, and trouble sleeping [8, 9].

The National Comprehensive Cancer Network (NCCN) guidelines for NETs recommend octreotide long-acting release (LAR) or lanreotide depot for first-line treatment in patients with metastatic NETs and carcinoid syndrome [10]. Both somatostatin analog (SSA) treatments are administered every 4 weeks [10], but they differ in formulation and administration. Lanreotide depot is a semi-solid, gel-like formulation in a pre-filled syringe, which is administered via deep subcutaneous injection [11], whereas octreotide LAR is a powder formulation requiring reconstitution which then forms microspheres and is administered intramuscularly [12]. Safety monitoring in the outpatient setting is similar for both SSA treatments [13].

Research has shown that the route of administration impacts treatment outcomes and patient experience among patients with NETs treated with an SSA. In a small qualitative interview study of US patients with NETs treated with a long-acting SSA and nurses who administer SSA treatment, patients expressed concerns about nurse training and familiarity with the injection process, proper injection preparation and administration, and inconsistencies with injection administration; nurses expressed challenges with octreotide injection preparation [14]. In addition, the efficacy of octreotide LAR appears to be vulnerable to administration effects related to its injection. A nursing quality improvement study conducted at the University of Texas MD Anderson Cancer Center found that up to 48% of octreotide depot gluteal injections failed to reach the intended muscle, which was associated with poor treatment outcomes such as increased flushing among patients [15]. Other patient-centric research has suggested that patients have a more favorable injection experience with lanreotide depot compared to octreotide LAR [16]. A study evaluating nurse preferences on SSA injection devices in the USA and Europe revealed that the most important syringe attributes were “confidence that the syringe will not be clogged” and “confidence that there is no loss of product during preparation or delivery” [17]. Compared with the octreotide LAR device, lanreotide depot scored higher on all attribute performance ratings, with the greatest differences in ratings for “fast administration from preparation to injection” and “confidence that the syringe will not be clogged.” Nearly all nurses (97.8%) expressed a preference for the lanreotide depot syringe compared with octreotide LAR [17].

The objective of this study was to describe SSA treatment experiences and preferences of patients with NETs using qualitative research methods. We aimed to describe drivers of patient transition from octreotide LAR to lanreotide depot for NETs treatment, explore the differences in treatment attributes between the long-acting SSAs as reported by patients, and elicit the SSA treatment attributes that are most important from the patient’s perspective.

## Methods

### Study design and participants

This was an observational, non-interventional, cross-sectional, exploratory, qualitative study of adult (≥ 21 years) patients in the USA with NETs who transitioned from octreotide LAR to lanreotide depot within the previous 12 months. Patients were eligible for this study if, at the time of screening, they self-reported having a clinician-confirmed diagnosis of NETs and had a minimum of 6 months’ treatment with each of octreotide LAR and lanreotide depot. Proficiency in the English language (i.e., ability to read, write, speak, and understand English well enough to complete informed consent process and take part in the interview process) and willingness to have their interview audio-recorded for the purpose of transcription and data analysis were also required. Patients with a history of brain metastases were excluded, but patients with a history of metastases in other sites were not excluded.

Participants were recruited through collaboration with the Carcinoid Cancer Foundation (CCF). All participants provided written informed consent. The study protocol was deemed exempt from institutional review board (IRB) oversight by Advarra IRB (Columbia, MD). The study abided by the Belmont Report, the Nuremberg Code, and the Declaration of Helsinki.

Data for this study were collected after patients’ self-reported treatment transition from octreotide LAR to lanreotide depot had occurred. Treatment-related decisions were determined by patients’ individual clinicians independently from this study.

Purposeful sampling was used to enroll 20 patients with NETs. In qualitative research, adequacy of a study’s sample size is largely justified based on achieving evidence of concept saturation (i.e., the point during data collection when no new concepts or relevant information are forthcoming) and additional interviews are unlikely to provide meaningful additions to the understanding of concepts under study [18]. Further details on saturation assessment are provided in analysis methods below.

### Qualitative interviews

Eligible participants completed a one-on-one, 60-min telephone interview with a trained qualitative interviewer using a semi-structured interview guide that explored their experiences with octreotide LAR and lanreotide depot. Telephone interviews were chosen to avoid restricting participation to limited locations with onsite interviewers, to prevent travel to clinic from interfering with patients’ ability to participate, and to allow sick patients to participate from the comfort of their own home.

The general purpose of the interviews was to provide a full understanding of the treatment attribute concepts that are relevant and important from the participant perspective; to conceptualize the treatment experiences and treatment burden of NETs; and to elucidate differentiating factors between NETs treatments. The interview began with broad exploratory questions on the participant’s overall experience with NETs and NETs treatment, then continued with focused open-ended questions for more in-depth exploration of topics such as the convenience of each SSA treatment, effectiveness in controlling symptoms, and treatment administration or injection complications. Participants were also asked about specific drivers of transition from one treatment to the other, their preferences for treatment, and rankings of product characteristics they deemed important. Follow-up probes were asked only as needed to gain a deeper understanding of a participant’s experience. Supplemental Table 1 presents a summary of the key discussion points and example questions included in the interview guide.

The interviews were conducted in a stepwise manner, analyzing data to allow for identification of emerging themes and concepts and areas for additional probing. All participant interviews were conducted by one of four research team members (CS, EH, RS, KM) who have experience with conducting qualitative interviews and are trained in qualitative data collection techniques. Prior to conducting interviews, all interviewers reviewed the study protocol and interview guide and were required to participate in mock interview sessions led by the qualitative research director. The mock sessions served to test the flow of the questions to find any problematic, slow, or awkward areas and to test the general timing of the interview.

### Qualitative coding and analysis

Interviews were audio-recorded, transcribed verbatim, and coded for qualitative content analysis using ATLAS.ti version 8 (ATLAS.ti Scientific Software Development GmbH; Berlin, Germany). The goal of the coding process was to identify relevant concepts and expressions of study participants and to organize these within similar groupings related to the interview guide. Through analysis of the data, conceptually equivalent codes were grouped and merged as appropriate based on themes and context identified in the transcript data. Consistency of coding was assured through assessment of inter-coder agreement. Transcripts were coded independently by 3 researchers and differences were resolved by consensus; a subset of 6 transcripts were co-coded by all 3 researchers. Treatment attributes for each SSA were coded as either favorable (i.e., product features that were spoken about in a positive way) or unfavorable (i.e., product features that were spoken about in a negative or critical way).

The qualitative dataset was evaluated for evidence of concept saturation to confirm the appropriateness of the sample size and ensure all information and themes were thoroughly elicited. Saturation assessment was conducted following guidance from the International Society for Pharmacoeconomics and Outcomes Research (ISPOR) Patient Reported Outcomes Good Research Practices Task Force and published literature [18, 19]. Given the initial sample size of 20 patients, the interview transcripts were organized chronologically and analyzed in 5 sets, with approximately 4 interviews in each set. To evaluate saturation, the appearance of novel concepts across these chronologically ordered sets was examined to determine the point at which no new information was obtained from interviews, which was indicative of the point at which saturation was achieved.

## Results

### Participant characteristics

**Fig. 1:**
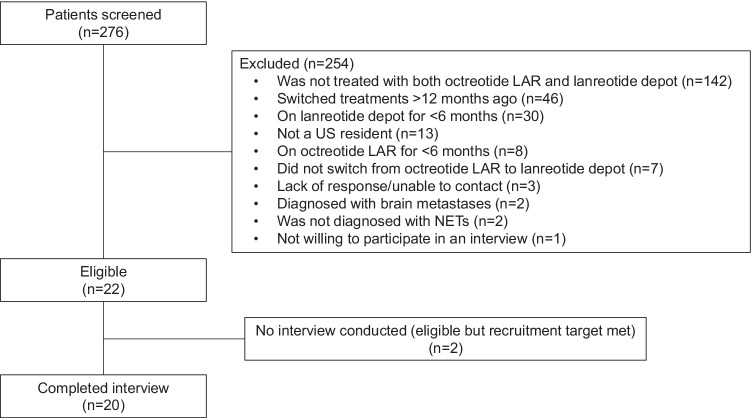
Patient flow diagram. Abbreviations: LAR, long-acting release; NETs, neuroendocrine tumor; US, United States

Of the 276 patients screened, a total of 20 participants completed interviews and were included in the analysis (Fig. 1). The first interview occurred on November 14, 2019, and the last interview occurred on March 19, 2020. The mean participant age was 58 years, 90% of participants were female (*n* = 18), and 85% were white (*n* = 17) (Table 1). Three-quarters of participants had GEP NETs (*n* = 15), 80% (*n* = 16) experienced carcinoid syndrome symptoms (e.g., gastrointestinal symptoms and flushing), and 65% (*n* = 13) had metastases in areas other than the brain.

**Table 1:** Demographic and health characteristics

	All Participants (*N* = 20)
Age, mean (SD), years	58.0 (10.3)
Female, n (%)	18 (90)
Origin of tumor, n (%)	
Gastrointestinal	12 (60)
Lung	4 (20)
Pancreas	3 (15)
Other	1 (5)
Hispanic, Latino, or Spanish origin, n (%)	1 (5)
Race, n (%)	
White	17 (85)
Black or African American	1 (5)
Other	2 (10)
Time since NETs diagnosis, years	
Mean (SD)	8.1 (4.2)
Median (range)	6.8 (2.0–17.8)
Treatment duration on octreotide LAR, n (%)	
6–11 months	1 (5)
12–17 months	2 (10)
18–23 months	3 (15)
≥ 24 months	14 (70)
Treatment duration on lanreotide depot, n (%)	
6–11 months	14 (70)
12 months	6 (30)
Reasons for transition, n (%)^a^	
Doctor recommendation	14 (70)
Treatment not working as expected^b^	11 (55)
Type of injection	9 (45)
Reaction at injection site	6 (30)
Side effects	2 (10)
Formulation of medication	2 (10)
Recommended by NETs patients	2 (10)
Injection setting	1 (5)
Changes in insurance coverage	1 (5)
Other	1 (5)

*LAR*, long-acting release; *NETs*, neuroendocrine tumors

^a^Participants could select more than 1 reason for transition in treatments

^b^This phrase was used in the eligibility screening form. It was open to further interpretation by patients during interviews and may encompass various reasons why patients did not believe that their treatment was working, such as spreading tumors or return/worsening of symptoms

Treatment duration varied between the 2 SSAs: most patients had received octreotide LAR for 18 months or longer (85%; *n* = 17), whereas all interviewed participants had received lanreotide depot for 12 months or less (100%, *n* = 20). The most common reasons for transition from octreotide LAR to lanreotide depot included recommendation from the doctor (70%; *n* = 14), treatment not working as expected (55%; *n* = 11), and injection type preference (i.e., intramuscular vs deep subcutaneous; 45%; *n* = 9).

### Saturation

A total of 34 unique favorable attribute concepts and 82 unique unfavorable attribute concepts were identified during interview transcript coding. For both the unfavorable and favorable groups of treatment attributes, more than half of all concepts (64.7% for each group) first appeared within the initial 8 interviews (interview sets 1 and 2). Although new unfavorable and favorable treatment attribute concepts were identified in the last set of interviews, upon further review, these concepts were not deemed to be unique. For example, the 3 unfavorable treatment attributes (lack of appetite, diarrhea, and nausea after injection) reported in the last set were reported by the same participant. Those same 3 concepts were previously reported by other participants in earlier interview sets as either disease symptoms or side effects related to other therapies. The one favorable treatment attribute (improvement in sleep) reported in the last set of interviews was reported by a single participant, who attributed the improved sleep to better symptom control—a concept that appeared/emerged within the first set of interviews.

## Experience with SSAs

Participant quotes regarding their experiences with octreotide LAR and lanreotide depot are presented in Table 2.

**Table 2:** Representative participant quotes regarding experience with SSAs

SSA Topic	Participant Quotes
Important SSA treatment characteristics	Again, hopefully they *help with bathroom issues, could alleviate some of the gas*—although I don’t know if anything can help with that. And that too – you know, *keep them from growing or help shrink them.* I’m sure that’s everybody’s hope. – Participant 00–01
	Well, to be honest, the *most important thing to me is that a treatment works*—and it works *with as minimal side effect as possible***.** That, for me, is my priority**.** *I don’t mind being inconvenienced for location, travel, going – you know, as long as it’s reasonable.* I don’t even mind some soreness or some side effects, because I know that a lot of medications have certain side effects. – Participant 00–24
Favorable attributes of SSA treatments	I was thrilled, because *it took away the pain and the nausea and the vomiting.* It didn’t cure the diarrhea itself, *but it took away the symptoms that made me not sure that I could go on one more day, and that made all the difference for me.* I can be homebound….I can do what I need to do if I’m just not in the bathroom with my head in a garbage can throwing up and doubled over in pain. And Sandostatin did that for me. – Participant 00–25
	*My bowel movements and diarrhea seem like they're more in control.* I'm thankful that I'm on lanreotide now. – Participant 00–16
Unfavorable attributes of SSA treatments	Regarding octreotide LAR: The burning. *It was a burning pain*….That happened one time. And I only had to do that once, but I remember driving home from work. I just couldn’t get home fast enough. I was – it was on fire. And I just had to keep ice on it that whole night and… **–** Participant 00–03
	Regarding lanreotide depot: Sometimes – it’s not always the same person giving it to you, *so it tends to work better when it is the same person,* but I realize people leave jobs and move or whatever. So right now, where I'm going for the shot is a new nurse, but she’s – seems to have gotten a hold on how to give it and *so my last two shots, which were from her, have been OK. No knots, no pain…* And I always know where it’s supposed to go too – which side, because *they switch sides every month*, so I keep a log of that…. ***–*** Participant 00–20
Preferences for SSA treatments	There’s a couple reasons…*the biggest one is it’s already prepared, ready to go.* Seems like a pretty smooth injection. And *I had some really, really good months where I had very little symptoms,* like everything was managed really, really well, which – and it was kind of crazy when I would have these spikes of symptoms. But for the most part, I have to say the symptom management was great. *The injections don’t really hurt. I don’t get painful lumps. It pretty much goes smoothly. I have no problems with it at all.* I haven’t had any side effects. – Participant 00–06 I think just the likelihood of successful injection, and it’s less – there’s *less steps for a successful – you don’t need to be shaking it or mixing it or – you know, it comes in a pre-filled syringe, and it takes the nurses* – you know, just pick a good location and one injection, and it’s done – and out the door you can go. And then *no side effects afterwards*, really, as far as *like the painful lumps* and stuff that came with every shot with Sandostatin. Haven’t had it at the injection time with lanreotide at all, so. – Participant 00–12

*LAR*, long-acting release; *SSA*, somatostatin analog

### Important SSA treatment characteristics

**Fig. 2:**
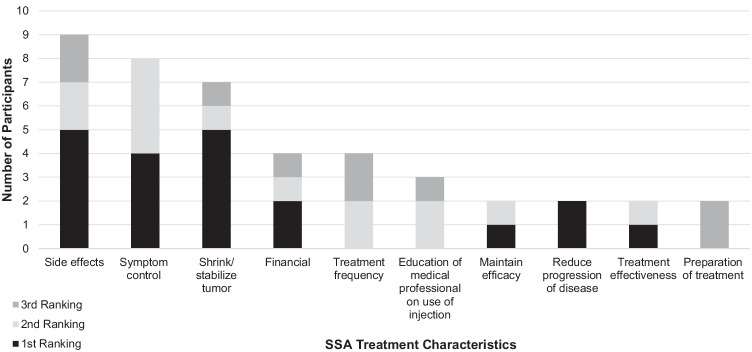
Top 10 important SSA treatment characteristics. Abbreviation: SSA, somatostatin analog

Control of symptoms (e.g., diarrhea, stomach cramping, and pain) was the most frequently expressed important characteristic of SSA treatment (90%; *n* = 18 participants), followed by ability to stabilize tumor growth (50%; *n* = 10). Following the discussion of the different types of characteristics, participants were asked to rank these characteristics from most important to least important. The top 10 important SSA treatment characteristics in rank order according to study participants are detailed in Fig. 2.

### Favorable attributes of SSA treatments

Participants reported 8 favorable attribute concepts for octreotide LAR and 26 favorable attribute concepts for lanreotide depot. Symptom control was the most frequently reported favorable attribute concept for both treatments (Table 3); however, the specific symptom(s) cited varied by participant (e.g., gastrointestinal symptoms, pain, energy/fatigue/tiredness, facial flushing).

**Table 3:** Top 3 most frequently expressed favorable and unfavorable attributes by SSA treatment

Frequency Ranking	Octreotide LAR (% of Patients Reporting)	Lanreotide Depot (% of Patients Reporting)
Favorable attributes		
1	Symptom control (60%)	Symptom control (65%)
2	Tumor stabilizing/shrinking (15%) Location of injection (15%) Frequency of injection (15%)	Less painful injection (45%)
3	No side effects (10%) Method of administration (10%)	No side effects (35%)
Unfavorable attributes		
1	Painful injection (65%)	Experience dependent upon administrator (35%)
2	Hard lump at injection site (60%)	Education needed on using needle (30%) Painful injection (30%) Hard lumps at injection site (30%)
3	Decreased efficacy over time (50%) Size of the needle (50%)	Size of the needle (25%)

*LAR*, long-acting release; *SSA*, somatostatin analog

### Unfavorable attributes of SSA treatments

Participants reported 43 unfavorable attribute concepts for octreotide LAR and 39 unfavorable attribute concepts for lanreotide depot. The most frequently reported unfavorable attribute concept was painful injection for octreotide LAR and experience dependent upon administrator for lanreotide depot (Table 3).

Many participants implemented coping mechanisms to alleviate some of the unfavorable side effects of SSA treatments. Nearly all participants (95%; *n* = 19) reported using coping mechanisms for octreotide LAR injections; the most common mechanism was using ice cubes or heating pads before or after injections on the injection site area (45%; *n* = 9). Most participants (70%; *n* = 14) reported using coping mechanisms for lanreotide depot injections; the most common mechanism was alternating sides for injections (25%; *n* = 5).

### Preferences for SSA treatments

When asked about their treatment preferences, the majority of participants (90%; *n* = 18) stated a preference for lanreotide depot over octreotide LAR. The most frequently reported reasons for preference of lanreotide depot included fewer side effects (25%; *n* = 5), better symptom control (25%; *n* = 5), fewer steps required for preparation (20%; *n* = 4), and less painful injections (20%; *n* = 4).

### Conceptual model

**Fig. 3:**
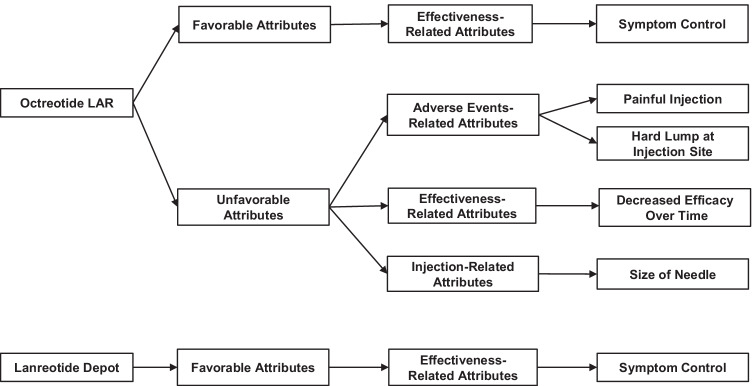
Conceptual model of key SSA treatment attributes^a^. Abbreviations: LAR, long-acting release; SSA, somatostatin analog. ^a^ Conceptual framework includes concepts endorsed by 50% or more of participants during qualitative interviews. No unfavorable attribute concepts for lanreotide depot were endorsed by 50% or more of participants

A conceptual model of key attributes of SSA treatments for NETs was developed based on the results of the qualitative interviews and included concepts expressed by 50% or more of the interview participants (Fig. 3). Symptom control was the key favorable attribute concept for both SSA treatments. Key unfavorable attribute concepts for octreotide LAR were painful injection, hard lump at injection site, decreased efficacy over time, and size of needle. No unfavorable attribute concepts for lanreotide depot were endorsed by 50% or more of the study participants.

## Discussion

This non-interventional qualitative study explored the treatment experiences and preferences of patients with NETs who had transitioned from octreotide LAR to lanreotide depot. Interview participants reported that the top 3 most important SSA treatment characteristics were side effects, symptom control, and ability to stabilize tumor growth. These characteristics aligned with what the participants reported as unfavorable and favorable treatment attributes. The interview participants, who had all transitioned from octreotide LAR to lanreotide depot, expressed more unfavorable attributes associated with octreotide LAR. Specifically, participants commonly reported experiencing painful injection, hard lump at injection site, decreased efficacy over time, and found the size of the needle to be problematic. These concepts were also noted for lanreotide depot but were reported less frequently. To cope with some of the unfavorable attributes reported (e.g., size of the needle, hard lump at injection site), the participants implemented various coping mechanisms (e.g., ice cubes/hot pads, shifting their body weight during injection). For both treatments, participants indicated that they experienced some range of symptom control.

Our findings were consistent with what has been reported previously regarding aspects of the injection experiences reported by patients with NETs. In a previous cognitive debriefing interview study for a draft SSA injection satisfaction survey, patient participants (*N* = 8) expressed confidence in their “usual” or familiar injection nurse (e.g., minimal or familiar/expected pain/discomfort), but expressed concerns about nurse training and familiarity with the injection process [14]. In our study, participants also mentioned how variability of the person administering the injection from month to month plays a role in the patients’ injection experience, and therefore symptom control. Participants ranked the need to educate medical professionals on the use of the injection as among the top 10 most important SSA treatment characteristics. Additionally, the top 2 unfavorable attributes for lanreotide depot were also related to administration, namely that the treatment experience was dependent upon the administrator and that providers needed education on using the needle. The coping mechanisms reported by participants in our study were also consistent with the discomfort alleviation tactics reported by Darden et al. (e.g., use of ice cubes/hot packs on injection site) [14]. Our results are also consistent with the findings of a recent systematic literature review (SLR) that explored treatment characteristics impacting patients’ and/or healthcare providers’ perspectives of lanreotide depot and/or octreotide LAR for NETs treatment [20]. In this review, technical problems with injections and associated pain, emotional quality/anxiety of injections, time and convenience of treatment administration, and independence were identified as factors that potentially impact SSA treatment experience. However, it is important to note that the Darden et al. study examined patients’ and nurses’ SSA treatment satisfaction, not treatment preferences, and the Cella et al. SLR noted that most studies on lanreotide depot and/or octreotide LAR treatment experiences did not explicitly aim to investigate treatment preferences [14, 20].

We believe our findings have implications for both clinical and health economics and outcomes research (HEOR), as well as real-world practice implications. Our results help address the evidence gap for understanding the experience of SSA treatments from the perspective of patients with NETs. Previous research has shown that a majority of patients prefer to participate in decision- making, particularly in oncology, and that preference has increased over time [21]. The US Food and Drug Administration (FDA) and other US policy makers encourage the incorporation of patient preferences into regulatory decision making and the drug development process [22, 23]. Our results, including the conceptual model developed through qualitative patient input, provide key insight on preference-related domains that can be used in future empirical research employing methods such as discrete choice experiments (DCEs) and multicriteria decision analysis.

Our findings could also help inform real-world clinical practice from multiple perspectives. The patient preference data from our study may be especially relevant to physicians who do not treat many patients with NETs and could enable patients and physicians to have better-informed discussions regarding SSA treatment, which could facilitate shared decision making for treatment planning and care. Our results could also be used to educate nurses or other healthcare providers who administer SSAs about the importance of proper administration, as well as provide tips they can share with patients to make the experience of receiving the injection as comfortable as possible both during and after the injection.

Our findings highlight the importance of disease and symptom control while balancing adverse events among patients with NETs treated with long-acting SSAs. The study also uncovered attributes linked to mode of administration of long-acting SSAs that impacted interviewed patients’ perceptions and preferences related to their overall treatment experience. However, this exploratory study has several limitations. Although evidence of concept saturation was observed in the qualitative dataset, caution should be taken when interpreting results due to the small sample size (*N* = 20). In addition, the study sample was limited to patients with NETs who transitioned from octreotide LAR to lanreotide depot and did not include equal numbers of patients who transitioned in the opposite direction. Therefore, patients who prefer lanreotide depot are likely to be overrepresented in our study sample. Specifically, for the SSA preference results, participants who transitioned to lanreotide depot may be more likely to prefer lanreotide depot or have an underlying preference causing them to initiate the treatment switch, so it may be most informative to focus on the specific qualitative reasons provided by patients in establishing their SSA treatment preference. In addition, participants’ time on lanreotide depot was artificially influenced by our eligibility criteria, which effectively excluded those on lanreotide for longer than 12 months, since the protocol required their transition to occur within the last year. Therefore, the finding that participants more frequently reported decreased efficacy over time as a negative attribute of octreotide LAR than lanreotide depot treatment may be due to most participants reporting ≥ 24 months of octreotide LAR treatment compared with ≤ 12 months of lanreotide depot. Although women comprised a slight majority of NETs cases (51.4–52.7%) in previous analyses of US epidemiological data [4, 24], they were overrepresented in our study sample, which was 90% female. Our study sample was also 85% white, which is similar to the US Cancer Statistics database analysis (77.2%) [24]. Although our research team strived to recruit more male participants, previous research has documented the difficulty in recruiting male participants for online health research as well as the overrepresentation of white women in such samples [25–27]. Finally, all participants were recruited through collaboration with a patient advocacy community, which may have an impact on the study results.

## Conclusion

The data collected from this study provide valuable insight for today’s patient-focused healthcare environment into the treatment attributes that patients with NETs consider important when making SSA treatment decisions. Specifically, these findings suggest that factors related to administration of injection are important to patients, alongside side effects and symptom control, and should be included in patient-provider communications in real-world clinical contexts. The results can also be used to generate testable hypotheses for future DCE studies and other direct assessments of treatment preferences, as well as other empirical evaluations of the real-world comparative effectiveness of SSA treatments in patients with NETs.

## Supplementary Information

Below is the link to the electronic supplementary material.

Supplementary file1 (DOCX 45 KB)

## Data Availability

Where patient data can be anonymized, Ipsen will share all individual participant data that underlie the results reported in this article with qualified researchers who provide a valid research question. Study documents, such as the study protocol and clinical study report, are not always available. Proposals should be submitted to DataSharing@Ipsen.com and will be assessed by a scientific review board. Data are available beginning 6 months and ending 5 years after publication; after this time, only raw data may be available.
