# Supplementary material for: A qualitative study to understand the experience of somatostatin analog treatments from the perspective of patients with neuroendocrine tumors
Source: Support Care Cancer. 2022 Apr 27;30(7):6307–16. doi: 10.1007/s00520-022-07054-x (PMC9135819; doi:10.1007/s00520-022-07054-x)
Supplement: Supplementary file 1 — Supplementary file1 (DOCX 45 KB) [file 520_2022_7054_MOESM1_ESM.docx]

SUPPLEMENTARY MATERIAL

Supplemental Table 1 Overview of Interview Sections and Example Discussion Questions

| **Section** | **Example Questions^a^** |
| --- | --- |
| Overall experience with NET | - What has it been like for you since you were diagnosed with neuroendocrine tumors? - What symptoms have you experienced *due to your neuroendocrine tumors*? - Which symptom is the most bothersome to you (related to your neuroendocrine tumors or carcinoid syndrome)? |
| Experience with octreotide LAR | - How was it decided to start your SANDOSTATIN^®^ treatment?   - PROBE: Who chose this treatment (e.g., doctor, you, or collaboration)?   - PROBE: What were the deciding factors?   - PROBE: What other treatment options were you given? - Can you describe your experience with SANDOSTATIN^®^?   - PROBE: What would you say is the most positive feature of the treatment? What do you like the most about it?   - PROBE: What would say is the most negative feature of the treatment, if any? - Did you feel that you experienced general improvement in your symptoms when receiving treatment with SANDOSTATIN^®^?   - PROBE: Could you explain why you felt this way? - On a scale of 0-10, where 0 would mean SANDOSTATIN^®^ did not help with the [symptom] at all and 10 would mean SANDOSTATIN^®^ was extremely helpful with the [symptom], how would you rate SANDOSTATIN^®^’s ability to help with the [symptom]? - Did you experience any side effects while on SANDOSTATIN^®^? - What are the aspects of SANDOSTATIN^®^ that make it tolerable to receive? - What are the aspects of SANDOSTATIN^®^ that make it difficult to receive? |
| Experience with lanreotide depot | - How was it decided to transition your treatment from SANDOSTATIN^®^ to SOMATULINE^®^?   - PROBE: What would you say was the primary reason for your decision to change therapy?   - PROBE: Who chose this treatment (e.g., doctor, patient, or collaboration)?   - PROBE: What were the deciding factors?   - PROBE: What other treatment options were you given? - Can you describe your experience with SOMATULINE^®^?   - What would you say is the most positive feature of the treatment? What do you like the most about it?   - What would say is the most negative feature of the treatment, if any? - Did you feel that you experienced general improvement in your symptoms when receiving treatment with SOMATULINE^®^?   - PROBE: Could you explain why you feel this way? - On a scale of 0-10, where 0 would mean SOMATULINE^®^ did not help with the [symptom] at all and 10 would mean SOMATULINE^®^ was extremely helpful with the [symptom], how would you rate SOMATULINE^®^’s ability to help with the [symptom]? - Do you currently experience any side effects while on SOMATULINE^®^? - What are the aspects of SOMATULINE^®^ that make it tolerable to receive? - What are the aspects of SOMATULINE^®^ that make it difficult to receive? |
| Treatment preferences | - Between SANDOSTATIN^®^ and SOMATULINE^®^, which treatment would you prefer? Why?   - PROBE: What are the characteristics of [participant’s response] that make you prefer that treatment? - What characteristics of neuroendocrine tumor treatment (i.e., SANDOSTATIN^®^/ SOMATULINE^®^) matter the most to you? Why?   - PROBE: How would you rank these characteristics from most important to least important? |

Abbreviations: LAR, long-acting release; NET, neuroendocrine tumor.

^a^ This table presents a sampling of questions from the 15-page interview guide.
